# Rank of the U. S Army Medical Corps

**Published:** 1874-07

**Authors:** 


### Editorial.

We announced in our last number that a Bill had been passed regulating the staff of the medical corps of the U. S. Army. We were at the time unacquainted with the provisions of the Bill, but supposed that it was the one which the medical profession, generally, had been urging Congress to pass. But we were mistaken in our supposition, as it differs materially from that Bill. The present bill, although ostensibly acquiescing to the demands of the profession, really cuts down the rank of the medical corps. In the place of five lieutenant-colonels, there are now two, and the Bill provides for fifty surgeons with the rank of major in the place of sixty as formerly.

The lesson to be learned from this, is that it is by personal effort with each member of Congress that anything is to be gained, and not by firing a lot of resolutions at the whole body. When each individual is made to see the justice of the demand, we may expect Congress, as a body, to perform that act of justice and not until then.

